# Second cancers after childhood cancer – GPs beware!

**DOI:** 10.3109/02813432.2013.824152

**Published:** 2013-09

**Authors:** A. J. Berendsen, A. Groot Nibbelink, R. Blaauwbroek, M. Y. Berger, W. J. E. Tissing

**Affiliations:** ^1^Department of General Practice, University of Groningen, University Medical Center Groningen, the Netherlands; ^2^Department of Pediatric Oncology, University of Groningen, University Medical Center Groningen, the Netherlands

**Keywords:** Childhood cancer survivors, general practice, general practitioner, long-term follow-up, second cancers, The Netherlands

## Abstract

**Background:**

One of the long-term effects in childhood cancer survivors (CCS) is the development of second cancers. In a cohort of CCS, this study describes how second cancers were presented, the way they were diagnosed, and the knowledge CCS had about their increased risk to develop a second cancer.

**Patients and methods:**

Selected participants were all adult five-year CCS (n = 1275) who were treated at the University Medical Center Groningen since 1965. Of these, 84 (6.6%) had developed a second cancer, of which 27 had died. The 57 survivors were asked to participate in a telephone interview.

**Results:**

Of the 57 CCS, 35 (61%) participated. Together they had developed 45 second cancers. Most participants (97%) were seen at the long-term follow-up clinic. Of all second cancers, 89% caused symptoms. Of all second cancers, the majority (56%) were first presented at the general practitioner's (GP's) office and 20% at follow-up testing. Of these CCS, only 28% were aware of their increased risk of developing a second cancer.

**Conclusions:**

It is important to inform CCS continuously regarding their increased risk, as a relatively small percentage are aware of this. Since most of these patients first reported their symptoms to the GP, all GPs should be aware of this increased risk, in particular because this concerns cancer at a younger age than normally expected. A survivor care plan might be an effective way of communication with both CCS and GPs.

- Childhood cancer survivors are at risk of late effects such as development of second cancers.
- Follow-up testing for late effects is advised in long-term effects clinics, but is still debated with regard to second cancers.
- Awareness is advised among GPs about the risk of second cancers as most are diagnosed by the GP.

## Introduction

In the Netherlands, each year about 550 children are newly diagnosed with cancer. Due to more effective treatment, the survival of childhood cancer has improved to an almost 80% five-year survival rate [1,2]. This improved prognosis implies a considerable increase in the number of survivors of childhood cancer [3].

Unfortunately, about two-thirds of adult childhood cancer survivors (CCS) suffer from long-term morbidity, due to long-term effects of the disease and its treatment [4,5]. Examples of associated health problems are cognitive damage, growth retardation, infertility, decreased cardiac function, endocrine insufficiency [6–12], and second cancers [13–15].

It is generally accepted that because of these late effects CCS need long-term follow-up; however, discussion continues regarding the best way to provide care for this group [16,17]. Risk classification in relation to the first cancer and received treatment is a guiding principle in this discussion [17]. Many medical centers decided to follow CCS in special long-term follow-up (LTFU) clinics. Also, because possible long-term effects comprise a large range of diseases a generalist, e.g. a general practitioner (GP), seems the most appropriate person to perform this type of follow-up for adult survivors [18].

One of the long-term effects in CCS is the development of a second cancer. In several cohorts risk ratios were six times higher as compared with the general population: the cumulative incidence was 3–4% after 20 years [13,19]. Risk factors are history of radiation therapy (usually ≥ 10 years after treatment) [13,19,20], history of chemotherapy (usually 3–5 years after treatment) [21–23], and an individual factor (gender, younger age at diagnosis, type of first cancer, genetic predisposition) [13,14, 22,24,25].

When discussing where the long-term follow-up of adult survivors with regard to second cancers should take place, it is important to know where and how the diagnosis of a second cancer was made. Therefore, we studied how the second cancers were presented, the way they were diagnosed, and the knowledge CCS had about their increased risk of developing a second cancer.

## Material and methods

### Patients

For this descriptive study we selected patients with a second cancer from all adult five-year CCS (n = 1275) who were treated at the University Medical Center Groningen (UMCG) between 1965 and 2005. According to the guidelines of the Dutch Childhood Oncology Group, patients are invited to visit the LTFU clinic (pediatric department) at varying intervals, once every 1–5 years. Some patients also receive follow-up testing from other specialists (e.g. neurologists after a brain tumor). All characteristics of these adults are known, including data on the first cancer, its treatment (e.g. surgery, chemotherapy – type and dosage, radiation therapy – field and dosage), complications and the occurrence of late effects, such as a second cancer. Our definition of a second cancer included not only malignant tumors but also benign tumors such as meningiomas and cerebral hemangiomas, as reported by others [25]. The selected adult CCS were contacted by letter.

### Structured interview

After providing informed consent, between April and September 2010 a structured interview was conducted by telephone by a researcher (AG). The questions were developed by a pediatric oncologist and a GP. To improve face and content validity, the concept questions were discussed with two senior researchers and piloted on three CCS visiting the LTFU clinic. The interview questions are listed in Table I.

**Table I.** Questions asked in interview with participants.

	Questions
Part 1	Who diagnosed the presence of your second cancer?
	Did you have any symptoms?
	If yes:
	• What symptoms did you have? • How long did it take before you told a doctor about your complaints? • How long did it take before a diagnosis was made after you had seen a doctor? • If there was a ‘delay’, what was the reason for this?
	If not:
	• How was the second cancer diagnosed? Please explain…….
Part 2	Did you know about the increased risk of a second cancer after childhood cancer?
	If so, who told you?
	Do you think it is important to have this information?
	If so, why?
	What is the best moment to receive this information?

In the first part of the interview patients were asked about the symptoms they had at the time the second cancer was diagnosed, how the diagnosis was made, and by whom. The period of time between the CCS first noticing symptoms and the first visit to a physician was defined as ‘patient's interval’. The period of time between the presentation at the physician and diagnosis was defined as ‘diagnostic interval’ [26]. Both intervals were categorized into six time periods (< week; 1 week–1 month; 1–3 months; 3–6 months; 6 months–1year; > 1 year). The second part of the interview included questions concerning the information given to the patient in the past about the risk of developing a second cancer.

Data were analyzed by descriptive methods (Mann–Whitney test), using SPSS. A p-value < 0.05 was considered significant.

## Results

### Characteristics

The median follow-up period for the entire cohort (n = 1275) was 28 years (range 8–44). Of the 84 (6.6%) patients with a second cancer, 27 had died. Of the remaining 57 CCS, 35 (61%) agreed to participate. Table II shows the characteristics of participants and non-participants. Almost all (34/35; 97%) participants were seen at the LTFU clinic with an interval range of 1–6/7 years. The 35 participants developed 45 second cancers. Median age at first cancer was five years while median age at second cancer was 27 years. Brain tumors were the most common second cancers, in particular meningiomas and hemangiomas.

**Table II.** Characteristics of the 35 participants and 22 non-participants and their second cancers (45/25).

	Participants (n = 35)	Non-participants (n = 22)
Male gender,^1^ n (%)	16 (46%)	7 (32%)
Follow-up time in years^1^ median (range)	29 (12–44)	25 (10–38)
Age at first cancer in years^1^ median (range)	5 (0–15)	7.5 (2–16)
Age at second cancer in years^1^ median (range)	27 (9–49)	27.5 (7–38)
Time (in years) since last visit to LTFU clinic n (%)		
< 1	14 (40%)	4 (19%)
1–2	3 (9%)	4 (19%)
2–3	4 (11%)	5 (23%)
3–4	3 (9%)	3 (14%)
5–6	7 (20%)	2 (0%)
6–7	3 (9%)	3 (14%)
Unknown	1 (3%)	1 (4%)
Second cancers	n = 45 n (%)	n = 25 n (%)
Brain tumor	18 (40%)	9 (36%)
BCC^2^	10 (22%)	6 (24%)
Melanoma	4 (9%)	0 (0%)
Breast cancer	2 (5%)	3 (12%)
Other^3^	11 (24%)	7 (28%)

Notes: ^1^No significant difference between participants and non-participants (Mann-Whitney test). ^2^BCC basal-cell carcinoma. ^3^For example, thyroid carcinoma, osteosarcoma, urothelial carcinoma, sigmoid carcinoma, tongue carcinoma, and ovarian cancer.

Of the second cancers, radiotherapy was (part of) primary treatment in 41/45 (91%). Of these 41 second cancers, 32 appeared in the previously irradiated area, mostly brain tumors (n = 17) and basal-cell carcinomas (BCC; n = 10). The nine second cancers which developed after radiotherapy, but not in the irradiated area, were melanoma (n = 2), brain tumor (n = 1), chondrosarcoma, sigmoid carcinoma, endometrial carcinoma, carcinoid, rectal cancer, and testicular cancer.

### Symptoms of second cancers

Of the 45 second cancers, five (11%) did not cause any clinical symptoms (Table III). The remaining 40 (89%) second cancers caused symptoms, most frequently skin changes, a palpable tumor, and neurological symptoms. Of the 18 brain tumors found, 14 (78%) caused neurological symptoms. All 10 BCC caused symptoms of itching skin, skin changes, poor healing, or cosmetically undesirable skin marks. The two breast cancers were palpated by the CCS themselves.

**Table III.** Symptoms and place of presentation of 45 second cancers (in 35 CCS).

			Place of presentation					
			Follow-up testing					
Second cancer	Symptoms		Follow-up testing total	Pediatric Oncologist (LTFU)	Other medical specialist	General practitioner	Specialist (visit for other reason)	Emergency department
	Yes	No						
Brain tumor	14	4	6	3	3	7	1	4
BCC	10	0	2	2	0	6	2	0
Melanoma	4	0	0			1	3	0
Mammary ca.	2	0	0			2	0	0
Thyroid ca.	1	0	0			1	0	0
Osteosarcoma	1	0	0			0	1	0
Other	8	1	1	0	1	8	0	0
Total	40 (89%)	5 (11%)	9 (20%)	5 (11%)	4 (9%)	25 (56%)	7 (16%)	4 (8%)

### Place of presentation

Second cancers were found at routine follow-up testing (LTFU clinic, or other medical specialist), GPs’ offices, medical specialists’ offices (visited by CCS for other health problems), or emergency departments.

At routine follow-up testing 9/45 (20%) of the second cancers were first presented (Table III). Of these, four (two BCC and two brain tumors) caused symptoms. Four brain tumors (benign) and a carcinoid caused no symptoms and were diagnosed while testing for local relapse of the primary cancer. Therefore, all five second cancers that did not lead to any symptoms were diagnosed at follow-up.

All 25/45 (56%) second cancers diagnosed by GPs caused symptoms: seven brain tumors and six BCCs, two breast cancers, one melanoma, one thyroid carcinoma, and eight other cancers. The two breast cancers were not actively screened because of the young age of the patient. Seven second cancers (16%) were diagnosed by medical specialists because patients visited them for other health problems. Four (8%) second cancers were diagnosed at the emergency department (all brain tumors).

### Interval in diagnosis

Data on patient's interval and diagnostic interval as reported by the participants are presented in Table IV. The patient's interval for BCC was one year or longer for 6/10 (60%). Diagnostic interval for brain tumors was short: 11/13 (85%) were diagnosed within one month.

**Table IV.** Patient's interval and diagnostic interval of symptomatic second cancers (n = 40).

Interval	Total n (%)	Brain tumors n (%)	BCC n (%)
Patient's interval			
≤ 1 week	14 (35%)	8 (57%)	
1 week–1 month	10 (25%)	3 (21.5%)	2 (20%)
1 month–3 months	4 (10%)		2 (20%)
3 months–6 months 6 months–1 year	2 (5%)		
≥ 1 year	10 (25%)	3 (21.5%)	6 (60%)
Total	40	14	10
Diagnostic interval			
≤ 1 week	14 (35%)	7 (50%)	2 (20%)
1 week–1 month	16 (40%)	7 (50%)	7 (70%)
1 month–3 months	4 (10%)		
3 months–6 months 6 months–1 year	5 (12.5%)		
≥ 1 year	1 (2.5%)		1 (10%)
Total	40	14	10

Notes: Patient's interval: time period between the CCS first noticing symptoms and first visit to a physician. Diagnostic interval: time period between presentation to a physician and the diagnosis.

Second cancers with a diagnostic interval of about three months were sigmoid carcinoma, cervical carcinoma, melanoma, and thyroid carcinoma. Rectal carcinoma, chondrosarcoma, melanoma, and testicular cancer involved a diagnostic interval of about six months, and one BCC was diagnosed (diagnostic interval) after more than one year.

### Patient's knowledge

Of the CCS, 10/35 (28%) reported they were aware of the increased risk of developing a second cancer. Of these, seven (70%) reported they were informed at the LTFU clinic. Most CCS 30/35 (86%) considered it important to be informed about this risk.

Reasons for better information (as reported by the CCS) were: knowledge causes greater alertness, reduces the shock at diagnosis of second cancers, and one should be informed about everything (Table V). Five participants thought it better not to be informed because information causes anxiety and revival of bad memories.

**Table V.** Participants’ opinion about receiving information (n = 30^1^).

Reasons for receiving information about increased risk of second cancers	Alertness	7 (23%)
	To be prepared/less shock at actual diagnosis of second cancer	7 (23%)
	Clarification/one should know everything possible	8 (27%)
	No reason	8 (27%)
Best moment to inform CCS of the increased risk	At the end of the treatment of primary cancer	7 (23%)
	After cure/when returning to the LTFU clinic	7 (23%)
	Do not know	16 (54%)

Note: ^1^Five participants thought it better *not* to be informed because information causes anxiety and revival of bad memories.

## Discussion

This study investigated the presentation of second cancers and the way they were diagnosed. We chose a structured interview to ensure that patients clearly understood all questions. Almost all participants (97%) were seen at the LTFU clinic. Of the second cancers, 56% were diagnosed at a GP's office and only 20% at follow-up testing. Remarkably, 89% of the second cancers caused symptoms. Patient's intervals were relatively long for BCCs. Only 28% of the CCS were aware of the increased risk of developing a second cancer.

In the present cohort the incidence of second cancers is 6.6% (median follow-up 28 years), which is comparable to other reports [14,21,22]. Radiotherapy was (part of) primary treatment in 91% of the second cancers; of these second cancers, 22% did not occur in the irradiated area. This implies that a number of the second cancers might not be caused by the therapy received. In our cohort the most common second cancers were brain tumors and most of these (78%) caused neurological symptoms. Physicians seem to be alert, since all brain tumors were diagnosed within one month after presentation. However, 21% of the CCS who developed a brain tumor had a patient's interval of more than one year, even though they had symptoms.

This first study on symptoms of second cancers and the way they were diagnosed in CCS is important, as it might affect our viewpoint concerning whether surveillance is needed for second cancers. Although one study suggested that specific screening for second cancers in CCS is necessary [14], we found that only 20% of second cancers were diagnosed at routine follow-up. Considering that almost all CCS (97%) were seen at the LTFU clinic, this percentage is not high. It is possible that the interval between the LTFU visits is too long to diagnose a second cancer in a timely manner. In the Netherlands, late effects surveillance is guided by national guidelines, with strict advice on how to perform follow-up. The interval of once every 1–5 years depends on diagnosis and treatment. Only breast cancer screening is advised as second cancer screening. However, in the present study, patients with breast cancer did not meet the criteria for screening since they were too young. The four asymptomatic brain tumors (meningiomas) were found by means of routine check-up when searching for local relapses of the primary cancer. However, brain tumors often cause symptoms and these CCS usually see a physician sooner or later. Also, there is no reason for surgery if a meningioma is asymptomatic. Therefore, follow-up testing for secondary brain tumors might not be indicated.

In our cohort, since most second cancers caused symptoms and were first presented to the GP, it is important that GPs are aware of the higher risk CCS have of developing a second cancer, even after many decades. Moreover, GPs might not expect some cancer types at this (younger) age.

Symptoms were often reported after a long patient's interval. This is probably (partly) due to the fact that only 28% of the CCS knew about their increased risk. One reason for this low percentage might be that the information was provided a long time ago; moreover, the information might not be remembered due to the patient's emotional state at that time. As suggested earlier, repeating the information and checking the knowledge obtained might be helpful [27]. CCS said they want to be better informed and this might reduce the patient's interval. A survivorship care plan made at the end of treatment and given to CCS and GPs might be an effective means of communication [18].

### Strengths and weaknesses

Little is known about how CCS with second cancers present to the healthcare system. This study is the first to examine the presenting symptoms and to describe where these patients are diagnosed.

We do realize that the study population is rather small. Besides, the retrospective nature of the study implies some recall bias that may affect the accuracy of the data (e.g. dates and presenting symptoms). However, given the very large percentage of second cancers not presented at follow-up testing, we feel that the conclusion of better knowledge for both patients and GPs is justified and important. Larger studies on this topic are warranted.

Not all second cancers might be known at the hospital and they might be underreported. This will not influence the present data, since we studied presentation and knowledge about second cancers and not, for example, the incidence.

In conclusion, only 20% of the second cancers were diagnosed by follow-up testing. Follow-up testing in special LTFU clinics seems less efficient: the interval between the LTFU visits might be too long to diagnose a second cancer in a timely manner.

It is important to educate CCS continuously about their increased risk, as only 28% of our study population were aware of this.

Because the majority of patients first reported symptoms to the GP, we think GPs should be aware of this increased risk as it concerns cancer at a younger age than one would generally expect [28].
